# Osteoporosis: A Step-by-Step Case-Based Study

**DOI:** 10.7759/cureus.23900

**Published:** 2022-04-06

**Authors:** Lokesh Goyal, Kunal Ajmera

**Affiliations:** 1 Family Medicine, Christus Spohn, Corpus Christi, USA; 2 Epidemiology and Public Health, Calvert Health Medical Center, Prince Frederick, USA

**Keywords:** osteoporosis, vertebral fracture, t-score, fall injury, geriatric hip fracture

## Abstract

Osteoporosis is a common disease that affects our elderly population. This disease usually gets undiagnosed for an extended period. Osteoporosis increases the risk of fracture in our elderly population and increases morbidity. The cost associated with osteoporosis does carry a substantial burden in our society. Here, we present a case of osteoporosis with a fracture diagnosed in clinical settings. We discuss different etiology, pathophysiology, and treatment options available to treat this medical condition.

## Introduction

Osteoporosis is a disease that causes a decrease in bone mass, increasing bone fragility and fracture [1]. Osteoporosis is a common disease, and it impacts one in three post-menopausal women and one in five men worldwide. There are roughly 200 million men and women who have osteoporosis in this world. The cost and morbidity associated with osteoporosis carry a substantial burden in our society. According to World Health Organization (WHO), a patient is diagnosed with osteoporosis if the Bone Mineral Density (BMD) T-score = -2.5 [1-3]. The test used to calculate the T score is also called the DXA scan. There are many instruments currently available that calculate the risk of fracture, one of them is called the Fracture risk algorithm (FRAX). FRAX calculates the risk of significant fractures (in ten years) like vertebral and hip fractures due to osteoporosis. FRAX can look at data provided by DXA and use it to predict the risk of fractures more accurately. In this case, we will focus on a patient who has osteoporosis. We will focus on etiology, pathophysiology and treatment options as well.

## Case presentation

**Figure 1:**
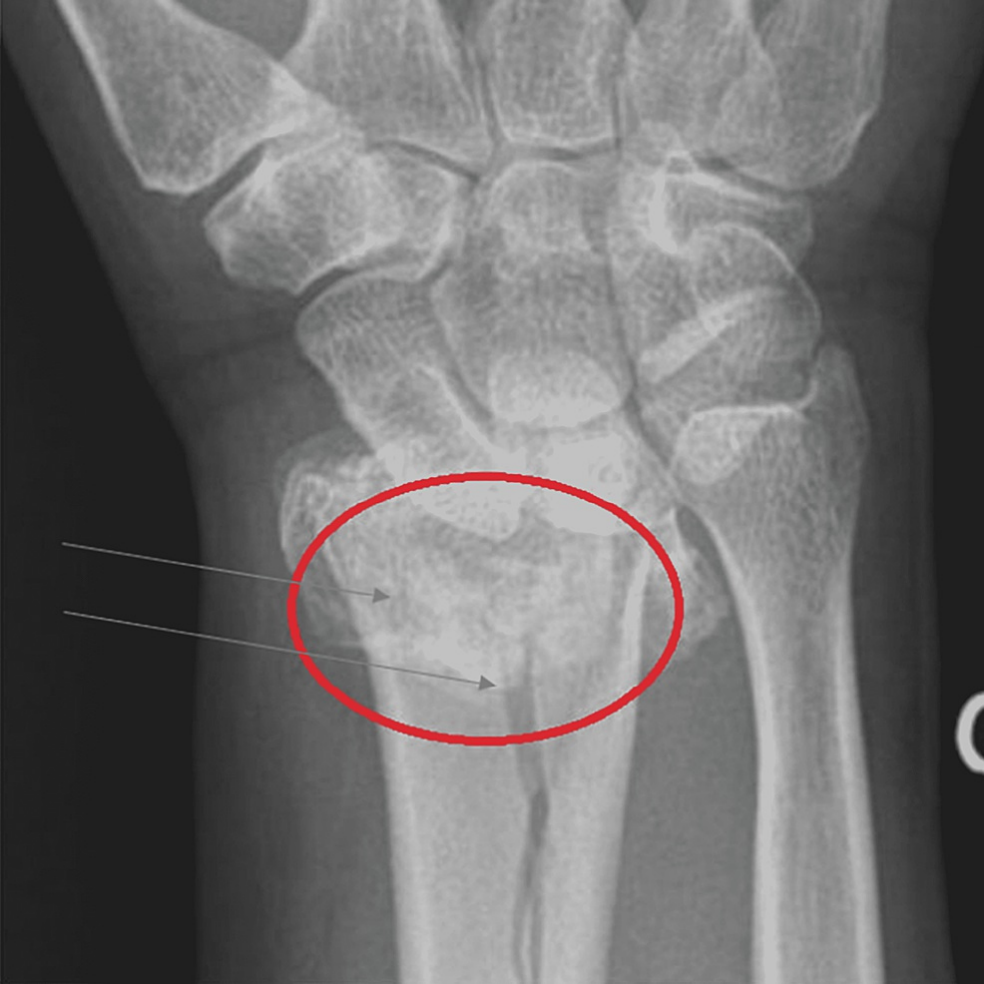
Colle's distal radial fracture

Patient X is a 62-year-old Caucasian female who presents to the outpatient clinic with right wrist pain and swelling following a fall on an outstretched hand in the garage at home. This patient has a past medical history of hypertension (HTN), chronic heart failure (CHF), pneumonia, chronic obstructive pulmonary disease (COPD), asthma, gastric ulcer, menopause (age 50), stooped posture, and vertebral bone fracture. The patient has a family history of CHF and HTN in her brother (age 55), a pelvic fracture in her mother (age 82), and the mother was also diagnosed with osteoporosis. The patient’s father has HTN. Patient X is married with four children and works in Walmart. She smokes one pack of cigarettes every day and occasionally drinks alcohol. She does no exercise and is fully mobile with no disabilities. The patient does not report symptoms of orthopnea, weakness, chest pain, palpitation, paroxysmal nocturnal dyspnea, or excessive bleeding. The patient is currently taking lisinopril for decreasing afterload; furosemide for reducing edema; atorvastatin for hypercholesterolemia; metoprolol for decreasing heart rate; omeprazole for stomach acidity; fluticasone for her asthma, and epidural steroid injection for lower back pain. Patient X reports to the clinic in acute distress and is oriented to time, person and place. The patient weighs 250 lbs., BP 145/88, pulse 90, and O_2_ saturation level of 92%. The patient lungs were clear on auscultation bilaterally. The cardiovascular exam showed a regular rate and rhythm without any murmurs. The right wrist is swollen and hurts to move. There was no edema on the left hand or feet. Radial, femoral, and dorsal pedis pulses were normal bilaterally. The patient was sent to the hospital to get an X-ray of the right hand (Figure 1), which revealed Colle’s fracture (distal radius) on the right hand. The hospital then puts a cast on the patient’s hand. The blood test was normal in this patient except for low levels of Vitamin D. The patient was also asked to get a DEXA scan, which revealed a T score of -2.9 (less than -2.5 is osteoporosis). The patient was started on bisphosphonates, raloxifene (selective estrogen receptor modulator [SERM]), and also given Vitamin D (1,000 mg) and calcium tablets. The patient was advised to start exercising daily and eat a healthy diet. She was also asked to be careful while walking.

## Discussion

Pathophysiology

The cause of osteoporosis [1-3] is an imbalance between bone formation and bone reabsorption. A typical bone is constantly being broken down and reformed. Around 10% of our total bone mass is under constant remodeling at any given time [4]. Due to menopause [5,6], the amount of estrogen secreted in a woman can decline rapidly [5,7]. The lack of estrogen [8] will increase the risk of bone reabsorption and decrease the deposition of new bone. Due to menopause, we also see an increase in basic multicellular units made of osteoclasts and osteoblasts cells. These osteoclast and osteoblast cells will sequentially resorb old bone and form new bone. This prolongs the osteoclast resorption time and relative shortening of the time for osteoblastic bone formation. The recent studies [6,9,10], both done in vitro and in vivo, show that, in the eugonadal state, estrogen will inhibit receptor activators of nuclear factor-κ B ligand (RANKL). RANKL [9,10] is a molecule found on the bone marrow stromal cells/osteoblast precursors and T and B cells. A decrease in the concentration of Vitamin D can also increase the risk of fracture and lower the BMD in the patient’s body. The primary source of Vitamin D comes from sunlight and diet. A severe deficiency of Vitamin D levels can lead to osteomalacia (in adults) or rickets (in children). These diseases cause softening of bones and increase the risk of fracture tremendously. The use of anti-acidity medications [2] like proton pump inhibitors (PPI) or H2 receptor blockers (Cimetidine) has been shown to increase fracture risk in adults. The increase in the risk of fracture due to anti-acidity medication is that these medications induce hypochlorhydria in the human body. This hypochlorhydria affects the absorption of calcium and therefore leads to a decrease in calcium in the body, increasing the risk of fracture. Any changes in sex hormones [8] are the most critical factor which affects bone loss due to aging; however, we still need to recognize the non-sex steroid hormonal changes that also occur in the human body. The most important hormone that affects bone physiology is the decrease in growth hormone secretion (as we age) from the pituitary gland. This decrease in growth hormone leads to a decrease in the production of insulin-like growth factors (IGF-1 and IGF-2) [4-6] from the liver. These hormones have a role in osteoblast activity and differentiation. A decrease in IGF is also associated with increased IGF inhibitory binding protein (IGFBP-2). An increase in IGFBP-2 in the human body leads to a decrease in BMD in adults [6-8].

Management

The most pivotal step in the diagnosis of osteoporosis is a DEXA scan (dual-energy x-ray absorptiometry). This test measures BMD. T score of less than -2.5 is considered a diagnosis of osteoporosis. Whereas a score of -1 to -2.5 is considered osteopenia. The NOF guidelines [4-6] state that a patient should undergo osteoporosis treatment not just after a hip/vertebral fracture or with a T- score≤−2.5, but treatment should also be considered in postmenopausal women and men with osteopenia (age > 50). The main goal of osteoporosis treatment is not just to increase BMD but also to prevent fractures in the future. Calcium and Vitamin D deficiency leads to an increase in the risk of bone loss and muscle weakness. This deficiency will, in turn, increase the patient’s risk of falling and fracture. By prescribing calcium and Vitamin D supplements to the patient, we can decrease fracture risk by 10%-15%. Multiple outcomes of the raloxifene evaluation (MORE) [6,7] study has shown that raloxifene, a SERM, reduces the risk of vertebral fracture by 30% if used continuously for three years. National Institute of Health and Care Excellence (NICE) [6,7] recommends using raloxifene in postmenopausal women at increased risk for osteoporosis or women intolerant of Bisphosphonates. Bisphosphonate is the class of drugs used for preventing osteoporosis. It has been the best choice for the treatment of osteoporosis since the 1960s. Bisphosphonates and their analogs bind at sites where bone resorption and new bone formation occur. The osteoclasts will ingest bisphosphonates bound to the mineral and therefore inhibit the function of osteoclasts. This will consequently lead to inhibition of bone resorption.

In addition to her past medical history and family history, these findings put this patient at risk of fracture due to osteoporosis. The past medical history of patient X (menopause, steroid meds, and old age) is consistent with the common risk factors for the development of osteoporosis. Patient X also has a history of vertebral fracture, which is most likely to have been caused due to osteoporosis. The most pertinent physical exam findings for this patient are the presence of stooped posture, history of vertebral fracture, and chronic back pain. The most critical risk factor for fracture in the case of osteoporotic patients is an unstable gait, which increases the risk of falls and, therefore, fractures. To rule out any risk of future fractures, this patient went through a thorough examination (gait abnormalities, orthostatic hypotension, and cognitive impairment). Patient X also went through a thorough neurological examination to rule out any spinal cord or peripheral nerves being compromised. This patient was prescribed bisphosphonates and calcitonin, which will help in inhibiting the osteoclast function. Raloxifene (SERM), this drug, will help decrease the risk of vertebral fractures and breast cancer in women. Vitamin D and Calcium supplements will help in increasing the BMD in the bones and decrease the risk of osteoporosis. The patient was also asked to have a good diet and exercise daily to encourage weight loss. The patient must do some weight-bearing exercise as this helps increase the BMD and helps decrease osteoporosis. Due to an increase in fracture risk from falling, the patient was advised to use a walker while walking.

## Conclusions

Through this case presentation, we realize that patients in our society are not appropriately screened for osteoporosis during their lifetime. This is usually due to a lack of medical knowledge among our patient population and sometimes the cost as well. Osteoporosis remains a public health problem and an economic burden to our society. As the incidence of osteoporosis continues to increase, it is clear that preventive interventions must be considered early on and sometimes as early as in utero. Patient education in primary care should focus on the benefits of a healthy lifestyle, a nutritious, and balanced diet (with Vitamin D and calcium supplements) in preventing the risk of osteoporosis. Patients must also avoid smoking, drinking, and illicit drugs as they have been shown to decrease the BMD and increase the risk of osteoporosis.
